# Extracellular vesicle-mediated preconditioning of osteoblasts by ER+ breast cancer cells: implications for the bone metastatic niche

**DOI:** 10.3389/fphar.2026.1844422

**Published:** 2026-09-28

**Authors:** De Shanna M. Johnson, Edward A. Shangin, Hannah L. Marrone, Elisabeth J. Richardson, Danielle O. Barkers-Woode, Caitlyn J. Collins, L. Monet Roberts

**Affiliations:** 1 Virginia Tech-Wake Forest School of Biomedical Engineering and Sciences, Virginia Tech, Blacksburg, VA, United States; 2 Department of Biomedical Engineering, Virginia Tech, Blacksburg, VA, United States

**Keywords:** breast-to-bone metastasis, estrogen receptor positive (ER+), extracellular vesicles, intercellular communication, molecular mechanisms

## Abstract

Breast cancer metastasis to bone most commonly occurs in late-stage disease and remains a major contributor to morbidity, reduced quality of life, and mortality, particularly in estrogen receptor-positive (ER+) tumors. Intercellular communication between tumor cells and bone-resident cells plays a critical role in promoting tumor invasion and development of metastatic bone lesions. Historically, tumor-bone interactions have been viewed largely through the lens of soluble factor-mediated signaling, particularly in the context of osteoclast-driven bone resorption and osteolytic lesion formation. However, the majority of bone metastases exhibit a mixed lytic and sclerotic phenotype, reflecting the combined involvement of osteoclast- and osteoblast-mediated remodeling. Here, we investigate how ER+ breast cancer-derived extracellular vesicle-enriched conditioned media influence osteoblast behavior and signaling *in vitro*, positioning osteoblasts as key intermediaries in bone metastatic progression. We first show that ER+ T47D cells release significantly higher EVs than MCF10A non-tumorigenic breast epithelial cells. Next, we demonstrate that osteoblasts actively internalize T47D-derived EVs by 24 h, resulting in significant alterations in their proliferation and signaling profiles associated with chemotaxis and vascular-associated pathways. Finally, we show that conditioned media from T47D EV-preconditioned osteoblasts did not significantly enhance breast tumor cell proliferation or overall motility outcomes. However, despite similar mean motility metrics between conditions, migration behavior exhibited distinct population-level distributions. Collectively, these findings demonstrate that ER+ breast cancer-derived EVs drive selective reprogramming of osteoblast signaling and function as an important component in tumor-bone niche conditioning.

## 1 Introduction

Breast cancer is the most diagnosed cancer and the second leading cause of death in women with global incidence continuing to rise (Sedeta et al., 2023; Giaquinto et al., 2024; Freihat et al., 2025). Estrogen receptor-positive (ER+) breast cancers are primarily associated with luminal molecular subtypes and collectively represent nearly 80% of female breast cancer cases (Bertucci et al., 2020; Wang et al., 2023). ER+ breast cancer frequently metastasizes to the bone, representing 70%–85% of all metastatic breast cancer cases (Wei et al., 2008; Bado et al., 2021; Nie et al., 2021; Wang et al., 2023; Huang et al., 2026). In addition to poor prognoses, bone metastases are often resistant to conventional systemic therapies due to the bone microenvironment functioning as a protective niche for disseminated tumor cells, highlighting a critical gap in effective therapeutic intervention (Sethakorn et al., 2022; Mittelman et al., 2026).

Dissemination of breast tumor cells to the bone niche disrupts homeostasis between osteoblasts, which are key bone-resident stromal cells that mediate bone formation, and osteoclasts (Feng, 2009), which drive bone resorption. This imbalance ultimately leads to metastatic lesions and substantial reductions in quality of life (Macedo et al., 2017; Bailey et al., 2019; Zajączkowska et al., 2019). Breast-to-bone metastases present with heterogeneous lesion types, of which 48% are osteolytic, 13% are osteoblastic (sclerotic), and 38% are mixed lesions (Li et al., 2014; Bailey et al., 2019). Osteolytic lesions are characterized by excessive bone resorption exceeding bone formation, which results in structurally fragile and porous bone that is associated with significant pain and a high risk of pathological fractures. In contrast, sclerotic lesions involve excessive bone formation relative to resorption; however, the newly deposited bone is often poorly mineralized and structurally abnormal (Bailey et al., 2019), conferring increased risk of fractures despite typically milder pain. Mixed lesions exhibit concurrent osteolytic and osteoblastic activity, producing regions of bone loss interspersed with areas of poorly mineralized bone tissue and clinical features characteristic of both lesion types. Collectively, these lesion phenotypes reflect profound dysregulation of osteoblast-osteoclast signaling within the metastatic bone microenvironment. Central to this dysregulation is the role of osteoblasts, which act as key coordinators of bone remodeling by regulating osteoclast differentiation and osteoclastogenesis through tightly controlled signaling pathways. In both primary bone malignancies and metastatic bone disease, perturbation in osteoblast-mediated control of osteoclastogenesis generates a permissive microenvironment that supports tumor growth and bone lesion formation (Sethi et al., 2011; Russo et al., 2022).

At the metastatic bone site, breast tumor cells further disrupt normal bone remodeling through extensive biochemical crosstalk with bone cells. This intercellular communication has classically been attributed to soluble factor signaling, including growth factors, cytokines, and chemokines that modulate osteoblast and osteoclast behavior (Kim et al., 2020; Sui et al., 2024). Tumor-derived factors, such as interleukins, transforming growth factor-β (TGF- β), and parathyroid hormone-related protein have been shown to alter osteoblast function and indirectly drive osteoclast differentiation and activation (Sethi et al., 2011; Wang et al., 2020; Russo et al., 2022), thereby promoting osteolytic bone destruction and highlighting osteoblasts as critical intermediaries linking tumor-derived signaling to lesion formation. While soluble factor-mediated signaling has been extensively studied, these alone do not fully account for the complexity of tumor-bone communication, particularly because these signals are transient and diffusible (Wei et al., 2025). Emerging evidence has recently implicated tumor-derived extracellular vesicles (EVs) as an additional, more sustainable mode of intercellular communication between tumor cells and osteoblasts in premetastatic niche formation (Loftus et al., 2020; Dai et al., 2021; Uehara et al., 2023). However, how ER+ breast tumor-derived EVs influence osteoblast behavior and downstream bone remodeling dynamics remain poorly understood.

*In vitro* studies have become increasingly important for dissecting the reciprocal signaling between breast cancer cells and bone-resident cells under controlled conditions. Although *in vivo* models have been instrumental in establishing the mechanisms underlying breast cancer dissemination and skeletal colonization (Mulcrone et al., 2017; Sowder and Johnson, 2018), these studies typically evaluate disease progression at experimental endpoints. As a result, they provide limited insight into the dynamic cellular and molecular interactions that occur during the earliest stages of metastatic niche formation. Understanding these interactions at the cellular level is critical, as communication between disseminated tumor cells and bone-resident cells influence tumor cell survival, adaptation, and subsequent colonization of the bone microenvironment.

In this study, we investigated how EVs derived from ER+ breast cancer cells modulate osteoblast (OB) responses *in vitro*. We first characterized EV production by T47D cells and OB viability and proliferation to establish optimal media conditions for subsequent co-culture studies. We then examined the uptake of T47D-derived EVs by OBs and resulting effects on OB soluble factor signaling, including inflammatory cytokines, chemokines, and vascular growth factors, in addition to OB proliferation. Finally, breast cancer cell motility was evaluated following exposure to conditioned media from OBs exposed to T47D-derived EV-enriched conditioned media to examine effects on tumor cell behavior. Overall, our findings demonstrate functional effects of T47D-derived EVs on osteoblast behavior and paracrine signaling during the earliest stages of metastatic progression.

## 2 Materials and methods

### 2.1 Cell culture and reagents

Human hFOB 1.19 osteoblast cells (OBs) (ATCC, CRL-3602) were cultured in DMEM/Ham’s F-12 media (Gibco 11039021) supplemented with 10% FBS (ATCC, 3020) and G418 at a final concentration of 0.3 mg/mL (Gibco, 10131035) per ATCC guidelines (osteogenic media). Human T47D breast cancer cells (ATCC, HTB-133) were cultured in RPMI-1640 medium (ATCC, 30–2001). Medium was also supplemented with 10% FBS (ATCC, 30–2020), and human recombinant insulin, zinc solution (Gibco; 12-585-014), at a final concentration of 0.2 U/mL (T47D media). Human MCF10A breast epithelial cells (ATCC, CRL-10317) were cultured in DMEM/F12 medium (ATCC, 30–2006) supplemented with 5% horse serum (ATCC, 30–2030), human recombinant epidermal growth factor (EGF; Gibco, PHG0311) at a final concentration of 20 ng/mL, hydrocortisone (Sigma-Aldrich, H0888) at 0.5 μg/mL, cholera toxin (Sigma-Aldrich, C8052) at 100 ng/mL, and human recombinant insulin (Gibco, 12-585-014) at 10 μg/mL. Cells were routinely tested for *mycoplasma* contamination and maintained under sterile conditions in a humidified incubator at 5% CO_2_, with hFOB 1.19 cells cultured at 34 °C and all other cell lines cultured at 37 °C.

### 2.2 Viability assay for selection of experimental media

To establish an optimal co-culture environment for both breast cancer and bone cells, a 2D cell viability assay was performed for 24, 48, and 72 h. OBs were seeded in a 24-well glass bottom well plate (Cellvis, NC1837310) at a cell density of 1 × 10^4^ cells/cm^2^. Viability for T47D cells was performed for 24 h at a cell density of 1 × 10^4^ cells/cm^2^. Cells were cultured under three media conditions: osteogenic media, T47D media, or 50%/50% mixture of the osteogenic and T47D media (tumor-bone basal media; TBBM). Following time-points, cells were washed with 1× phosphate buffered saline (PBS) and stained with a mammalian cytotoxicity kit (Invitrogen, L3224) for analysis. Five regions of interest (ROI) were imaged across each well. Images were acquired at 10× magnification using the Discover ECHO Revolution microscope.

### 2.3 Extracellular vesicle (EV) harvesting and enrichment for conditioned media conditions

T47D or MCF10A breast cells were plated at 1 × 10^4^ cells/cm^2^ in appropriate dishes and allowed to grow for 42 h. Cells were then rinsed with PBS then serum starved in either TBBM or MCF10A-osteogenic media (MCF10A control media) for an additional 6 h to eliminate serum-based EVs. EVs were harvested as previously described (Shurer et al., 2019). In brief, EV-enriched conditioned media from serum-starved cells was clarified by pelleting cellular debris through two consecutive centrifugations at 600 × g for 5 min. For EV co-culture with OBs, T47Ds and MCF10As were serum-starved in TBBM for appropriate experiments. Additionally, control TBBM also remained serum-free to prevent introduction of EVs present in serum.

### 2.4 Nanoparticle tracking analysis (NTA)

Clarified T47D or MCF10A EV-enriched conditioned media was analyzed using the Nanosight Pro (Malvern). Imaging was performed over 60 s with five captures per sample. Subsequent particle analysis was performed using the NS Xplorer software (Malvern) to quantify EV size distribution and concentration. Exosomes are defined as 50–150 nm and microvesicles (MVs) are considered 150–1,000 nm. EV production rate was calculated by normalizing total particle counts to the number of cells generating EVs and the duration of serum starvation. Specifically, particle concentration (particles/mL) was divided by the number of cells in EV-producing dishes and then normalized to the 6 h serum-free incubation period, yielding particles per cell per hour as a production rate. Maximum production rate was quantified by finding the maximum across the exosome and MV regimes.

### 2.5 EV uptake assay

OBs were seeded at 1.25 × 10^4^ cells/cm^2^ in a 24-well culture dish and allowed to establish a monolayer for 5 days. Following monolayer establishment, OBs were co-cultured in either T47D EV-enriched conditioned TBBM or naïve TBBM as a control. Conditioned media from OBs was collected every 6 h over a 24 h time period and EV-enriched clarified media was harvested and analyzed via NTA as described above to quantify EV uptake.

For fluorescence visualization of EV uptake, T47D cells were cultured in six T75 flasks until reaching 95%–100% confluency and serum-starved in T47D media for 24 h to generate EV-enriched conditioned media. Conditioned media was collected and centrifuged at 600 × g for 5 min to remove cellular debris, followed by filtration through a 0.45 μm filter. EVs were isolated by centrifugation at 16,000 × g for 1 h at 4 °C. Following centrifugation, the supernatant was decanted, and the EV pellet was resuspended in PBS for labeling. EVs were fluorescently labeled with MemGlow (Cytoskeleton, MG04) at a final concentration of 0.2 μM and incubated for 20 min at room temperature in Protein LoBind tubes (Eppendorf, 0030108116). Excess unbound dye was removed using a centrifugal ultrafiltration concentrator (Sartorius, VS02V2). Following purification, labeled EVs were recovered and resuspended in serum-free osteogenic media.

Osteoblasts were fluorescently labeled with CFSE (ThermoFisher, V12883) at a final concentration of 10 μM prior to EV exposure. Specifically, cells were incubated with CFSE for 15 min at 37 °C and subsequently exposed to MemGlow-labeled EVs for 24 h. Following EV incubation, the culture medium was aspirated, and cells were washed once with PBS. Cells were then incubated with an acid wash buffer consisting of 0.5 M NaCl and 0.2 M acetic acid (pH 3.0) for 30 s at room temperature to remove surface-associated EV fluorescence. The acid wash buffer was immediately aspirated, and cells were washed three times with PBS. Cells were fixed with 4% paraformaldehyde for 15 min at room temperature, washed three times with PBS, counterstained with DAPI (1:5,000 dilution), and imaged at 20× using an EVOS fluorescence microscope (ThermoFisher).

### 2.6 Multiplex growth factor/cytokine profiling

Conditioned media from T47D EV- and naïve-treated OB cultures was collected in Protein LoBind tubes, flash frozen and stored at −80 °C until used for multiplex growth factor and cytokine profiling analysis. Data acquisition was performed using the xMAP INTELLIFLEX® platform (Luminex Corp) at the University of Virginia Flow Cytometry Core. Analytes were organized across categories of inflammatory cytokines, chemokines, and vascular growth factors of interest across three panels as follows: Panel 1: IFN-γ, IL-β, IL-6, IP-10/CXCL10, TNF-α, and PDGF-AB/BB; Panel 2: 6Ckine/CCL21/Exodus-2, MIP-3β/CCL19, and SDF-1/CXCL12; and Panel 3: IL-8/CXCL8, VEGF-C, VEGF-D, VEGF-A, and FGF2. Concentration of analytes were analyzed and quantified using Belysa® Immunoassay Curve Fitting Software (MilliporeSigma).

### 2.7 Proliferation assay

OBs were either conditioned with naïve TBBM, T47D EV-enriched conditioned media, or MCF10A EV-enriched media and proliferation was quantified using the Click-iT™ EdU cell proliferation assay (Invitrogen, C10340) as previously described (Roberts et al., 2025). After 18 h of culture, 10 µM EdU was added to media and incubated for 6 h. Subsequently, cells were washed with PBS and fixed with 4% paraformaldehyde. Samples were then permeabilized with 0.5% Triton X-100 and stained with the Click-iT™ reaction cocktail per the manufacturer’s instructions. The cells were then counterstained with Hoescht 33342 at a concentration of 5 μg/mL. Once stained, five ROIs were imaged across each well under 10× magnification using the Discover ECHO Revolution microscope. A custom CellProfiler pipeline was used to identify and quantify DAPI-positive nuclei. EdU-positive nuclei were then detected using the same segmentation framework to evaluate the labeled cell population.

### 2.8 Motility assay

7.5 × 10^4^ T47D breast cancer cells were first labeled with CellTracker dye and subsequently embedded within a hydrogel composed of 1.8 mg/mL methacrylated collagen (PhotoCol) (Advanced Biomatrix, 5270) and 0.5 mg/mL growth factor-reduced basement membrane extract (R&D Systems, 3445-005-01). Gels were allowed to thermally polymerize and then photo-crosslinked using 405 nm light for 45 s. Prior to treating the T47D gels, OBs were preconditioned with naïve TBBM, osteoblast conditioned media (OBCM), and collected media from osteoblasts preconditioned with T47D EV-enriched TBBM (PCOB) for 24 h and then collected, respectively. After preconditioning, T47D-encapsulated gels were also cultured with either naïve TBBM, OBCM, or PCOB for 24 h. Constructs were imaged using an onstage incubator imaging system (EVOS M7000) at 37 °C and 5% CO_2_. Time-lapse imaging was performed for 6 h with images acquired every 15 min from 5 ROIs. Time-lapse images were tracked using Celleste, and cell migration metrics were quantified using a custom MATLAB-based analysis pipeline adapted from previously published methods (Roberts et al., 2022).

### 2.9 Image quantification

For viability assessment, images were analyzed using custom Python and MATLAB scripts to quantify positive staining for calcein-AM (live cells) and ethidium homodimer-1 (dead cells). Viability was reported as the ratio of live cells to total cells across ROIs. For proliferation, total nuclei were quantified using Hoechst 33342 or DAPI staining, and proliferating cells were identified through positive EdU staining. Proliferation was expressed as the percentage of EdU-positive cells relative to the total cell count across ROIs. Custom scripts and data can be provided upon request.

### 2.10 Statistical analysis

All statistical analysis was performed using GraphPad Prism 11.0.0 (Graphpad Software). Statistical significance was indicated by *p* < 0.05 and *post hoc* testing was performed only if significance was detected. For the viability assays and population-level motility assays, a one-way ANOVA was performed. For migration assay analyses at a single cell level, nonparametric tests were used due to the non-normal distribution of single-cell tracking data. Here, a Kruskal–Wallis test was used to assess overall differences across conditions, followed by Dunn’s multiple comparisons test for *post hoc* analysis. Finally, the Mann–Whitney U test was used for pairwise comparisons, and the Kolmogorov–Smirnov test was used to evaluate differences in distribution shape and population-level shifts in migration behavior. For pairwise comparisons, Welch’s t-test was used for the proliferation assay, while unpaired t-tests were used for EV production rate and Luminex assays to identify significant differences between groups. For multiplex cytokine analyses, raw p-values were reported followed by correction using the two-stage linear step-up Benjamini, Krieger, and Yekutieli method with a false discovery rate of 5%.

## 3 Results

### 3.1 T47D breast cancer cells exhibit enhanced extracellular vesicle release, particularly in the microvesicle regime

As key mediators of intercellular communication beyond soluble factor signaling, extracellular vesicles (EVs) are comprised of two major subtypes defined by their biogenesis pathways and size: exosomes and microvesicles (MVs) (Figure 1A). Exosomes are secreted from cells via the endosomal-multivesicular body pathway and traditionally described to be between 30 and 150 nm in diameter (Latifkar et al., 2019; Lin et al., 2020; Vučemilović, 2024). MVs are shed directly from the plasma membrane and typically span 150–1,000 nm in diameter (Muralidharan-Chari et al., 2010; Jeppesen et al., 2023). Our previous work demonstrated that breast cancer cells, including ER+ T47D cells (Pink et al., 1996; Yu et al., 2017), exhibit distinct plasma membrane morphologies compared to non-cancerous MCF10A breast epithelial cells (Shurer et al., 2019). Importantly, we further established that specific membrane features are closely associated with increased EV release corresponding to distinct size distributions in the MV regime. Building on these observations, we sought to profile EV size distributions produced by T47D cells and compare them with EVs derived from MCF10A cells as a non-cancerous control. Nanoparticle tracking analysis showed that EV-enriched conditioned media from both T47D and MCF10A cells largely possessed vesicle populations within 0–500 nm, consistent with expected exosome and MV populations (Figure 1B). NTA also demonstrated that T47D cells exhibit increased vesicle production compared to MCF10A cells. Specifically, T47D cells exhibited a higher maximum exosome production rate relative to MCF10A cells; however, this difference did not reach statistical significance (Figure 1C). In contrast, T47D cells displayed a significantly higher maximum MV production rate as compared to MCF10A cells (Figure 1D). Together, these results further support that T47Ds display enhanced EV production, driven predominantly by a significant increase in MV release.

**FIGURE 1:**
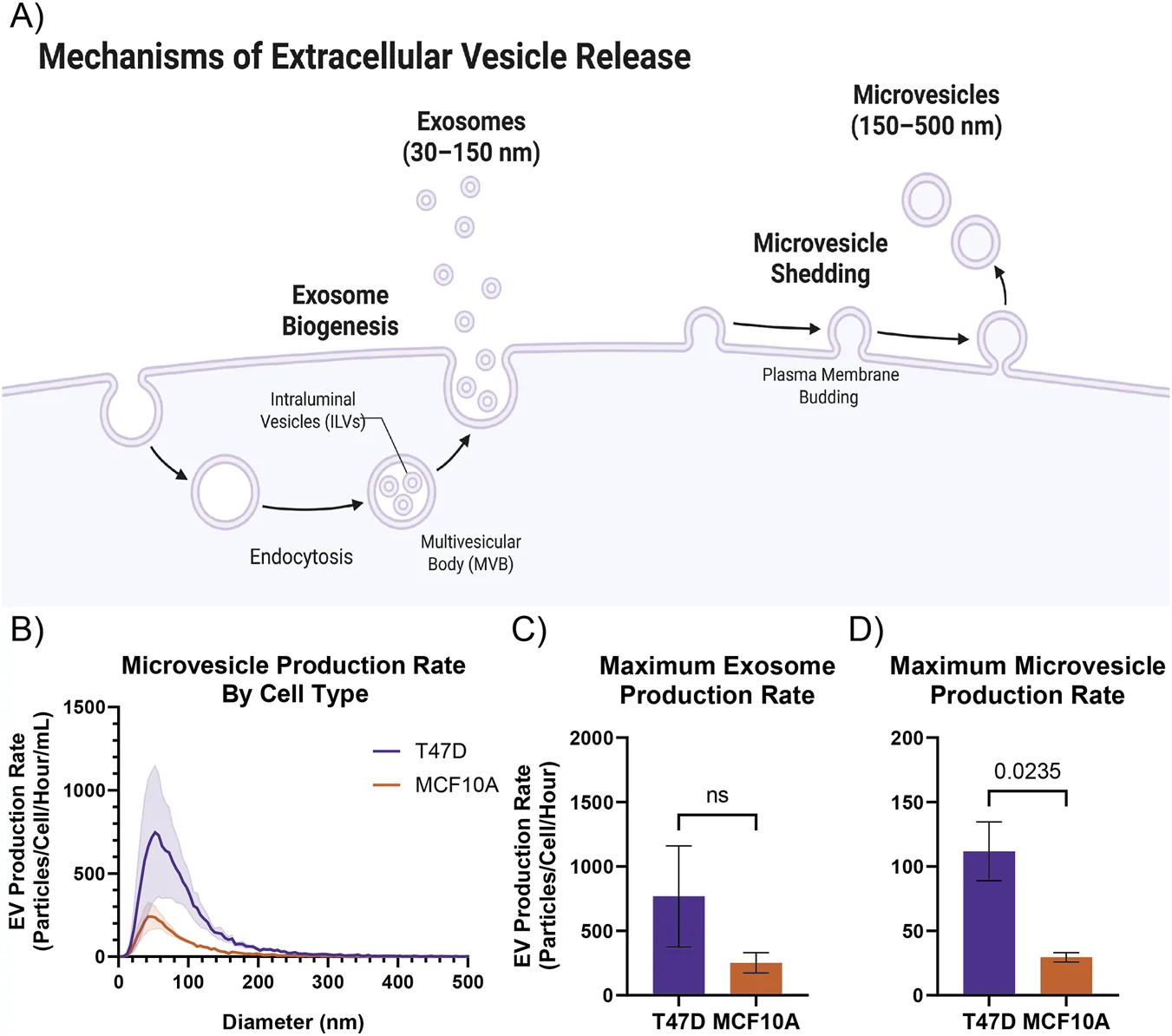
T47D cells exhibit enhanced extracellular vesicle production. **(A)** Schematic illustrating extracellular vesicle (EV) biogenesis pathways, including exosome release through the endosomal-multivesicular body pathway and microvesicle (MV) shedding via outward budding from the plasma membrane. Schematic adapted from Nashed, M. (2026) on BioRender. **(B)** Representative histogram from nanoparticle tracking analysis (NTA) showing EV production rate and size distribution of EVs for T47D breast cancer cells and MCF10A cells as a control (n = 3 per group). Particle concentration is normalized to the max peak for each experimental group. Shaded area shows the standard error of the mean (SEM) for all histograms. **(C)** Maximum production rate in the exosome regime (30–150 nm) for T47D and MCF10A cells. **(D)** Maximum production rate in the MV regime (shown here as 150–500 nm) for T47D and MCF10A cells. All data and error bars in the bar graphs represent mean ± SEM from n = 3 independent experiments. Statistical significance was determined using an unpaired two-tailed Student’s t-test to calculate represented *p*-values; n.s indicates not significant.

### 3.2 Culture condition optimization supports sustained viability of T47D cells and osteoblasts

The premetastatic niche is a complex environment that requires distinct nutrients and growth factors to support both breast cancer cells and bone-resident cells. To model culture conditions that allow both cell types to maintain proper function while physiologically representing the metastatic niche, we evaluated various media compositions at varying concentrations. OB viability was not affected when cultured in T47D nor TBBM (Figures 2A,B). Similarly, T47D cells displayed comparable viability when cultured in osteogenic media and TBBM (Figures 2C,D). Since no significant differences in cell viability were observed across the tested conditions (Supplementary Figure 1), TBBM was selected for all subsequent experiments to ensure experimental consistency and physiological relevance to the premetastatic niche.

**FIGURE 2:**
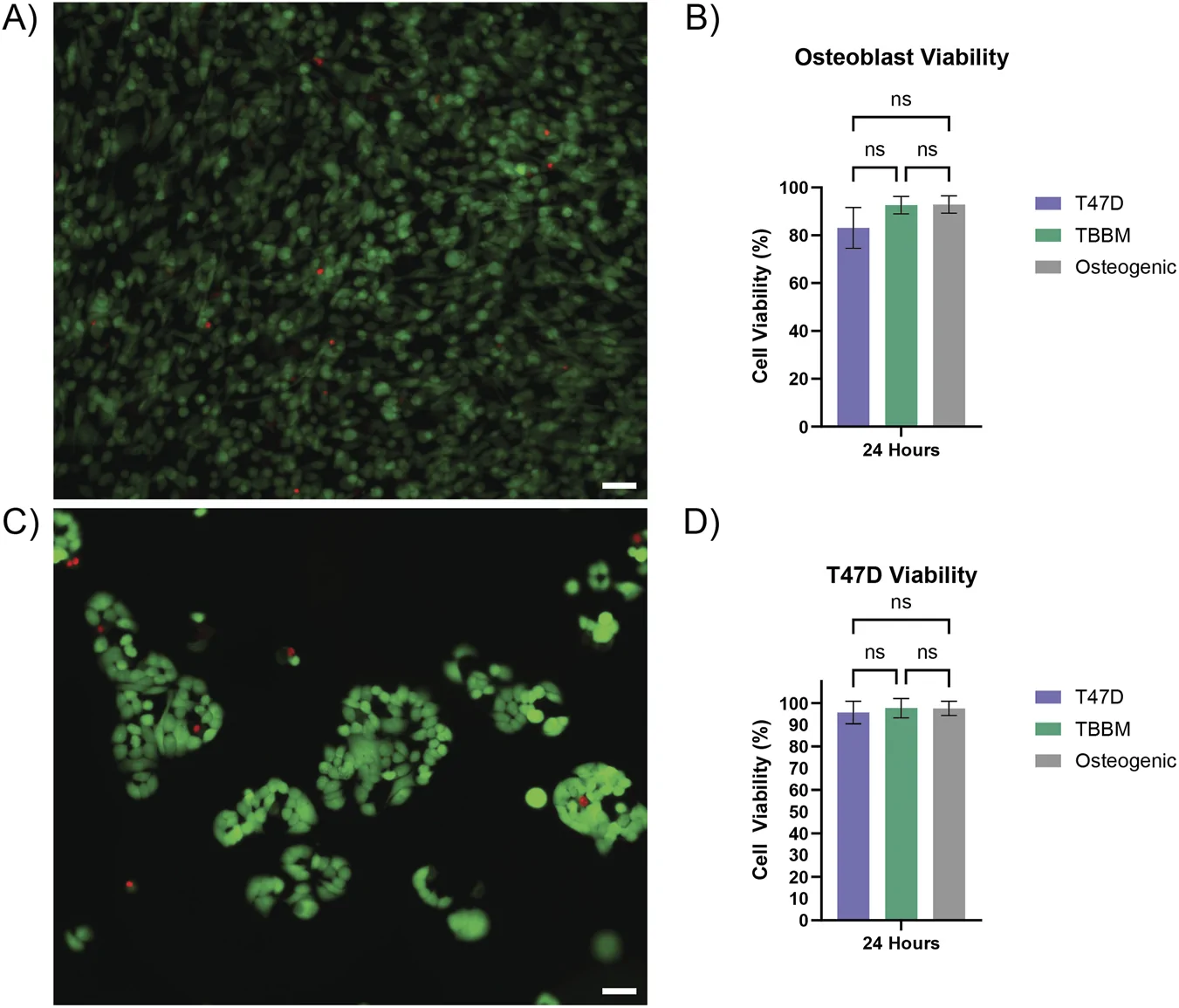
T47D and OB cell viability was supported in TBBM similar to normal culture media conditions. **(A)** Representative image staining of OBs cultured in TBBM after 24 h. Live cells are denoted in green and dead cells are denoted in red. Scale bar represents 50 µm. **(B)** Quantification of OB viability cultured in either T47D, TBBM, or osteogenic media at 24 h (n = 3). Statistical analysis was performed via one-way ANOVA. Live cells are denoted in green and dead cells are denoted in red. **(C)** Representative image of T47D cells cultured in TBBM after 24 h. Scale bar represents 50 µm. **(D)** Quantification of T47D viability media conditions at 24 h. All data and error bars in the bar graphs represent mean ± SEM from n = 3 independent experiments. Statistical analysis was performed via one-way ANOVA. n.s indicates not significant.

### 3.3 Time-dependent uptake of T47D-derived EVs by osteoblasts peaks at 24 h

Given accumulating evidence that EVs mediate long-distance communication and contribute to the establishment of a tumor-permissive microenvironment at secondary sites (Forder et al., 2021; Asao et al., 2023; Nidhi et al., 2025; Semeradtova et al., 2025), we next investigated how T47D-derived EVs interact with OBs, which are abundant within the endosteal niche adjacent to the bone marrow where metastatic tumor cells typically colonize (Cordeiro-Spinetti et al., 2015; Haider et al., 2020; Maurizi et al., 2026). We hypothesized upon arrival in the bone niche, BC-derived EVs are internalized by OBs, thereby reprogramming osteoblast function to support tumor cell colonization and growth (Figure 3A). Following co-culture of OBs with T47D-derived EVs over 24 h, a progressive time-dependent decrease in EV concentration was observed with the lowest levels detected at 24 h (Figure 3B). Based on this reduction, OBs at the 24 h time point were defined as preconditioned for subsequent experiments. To assess whether particle counts reflected *de novo* secretion by OBs in the total concentration, a control group of media harvested from OBs cultured in the TBBM alone was included (Figure 3C). Furthermore, fluorescent microscopy confirmed internalization of T47D-derived EVs following 24 h exposure (Figure 3D), corroborating the observed depletion of EVs from our NTA results and supporting the conclusion that OB preconditioning occurs by 24 h.

**FIGURE 3:**
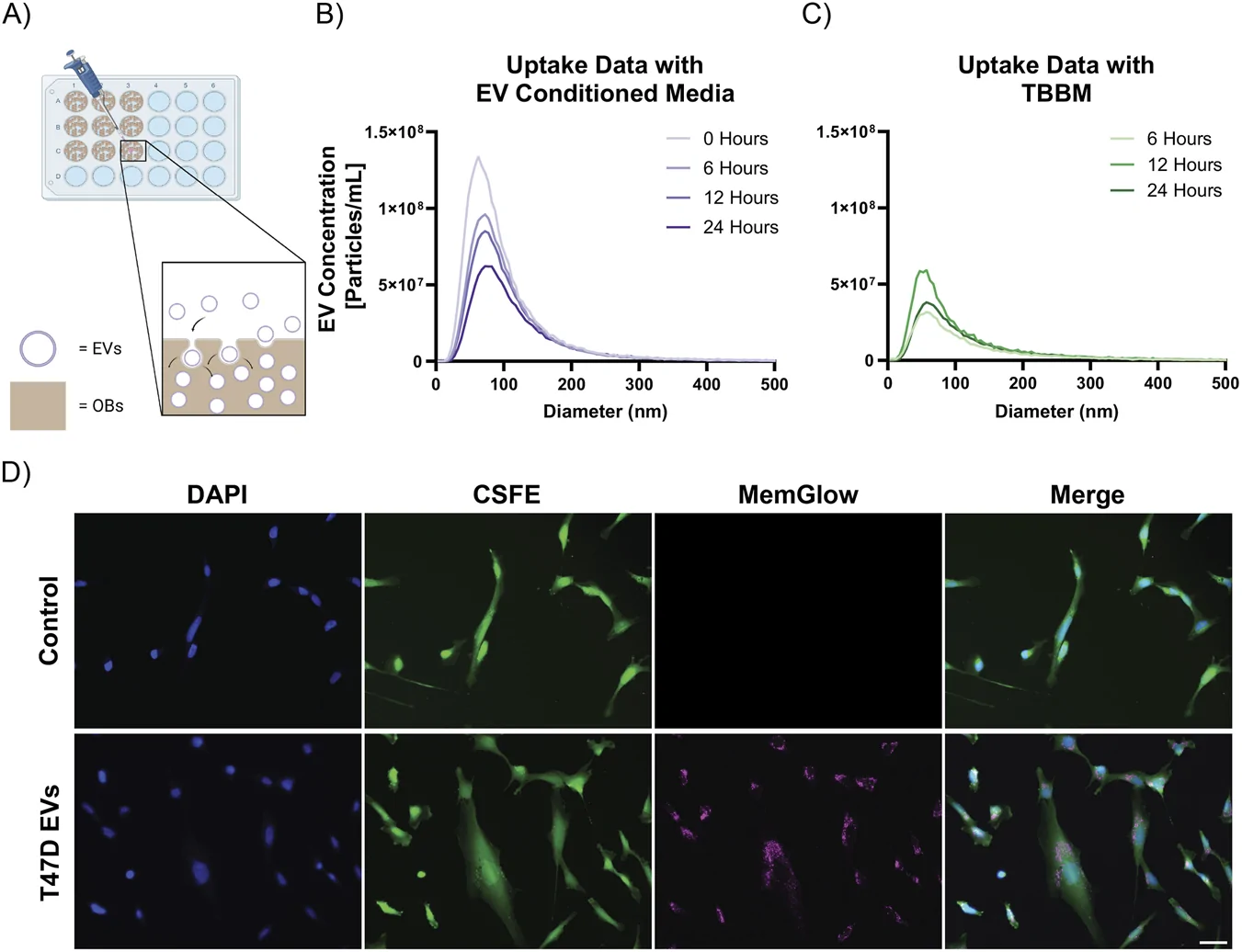
OB uptake of T47D EVs is highest at 24 h. **(A)** Workflow schematic illustrating OB exposure to T47D-EV preconditioning. **(B)** Representative NTA histogram of concentration and size distribution of EVs remaining in the culture media of T47D EV-treated OBs at 0, 6, 12, and 24 h (n = 3). **(C)** Representative NTA histogram of concentration and size distribution of EVs in the culture media of OBs cultured in TBBM at 6, 12, and 24 h (n = 3). **(D)** Representative images of T47D-EV uptake after 24 h with nuclei of OBs stained with DAPI (blue), the cytoplasm of OBs stained with CFSE (green), and pre-stained T47D EVs internalized within OBs stained with MemGlow (magenta). Scale bar represents 50 µm.

### 3.4 Osteoblast preconditioning increased secretion of pro-tumorigenic soluble factors and proliferation

Previous studies have demonstrated that OBs treated with murine mammary tumor-derived EVs exhibit increased cytokine production alongside reduced proliferation, metabolic activity, and functional capacity (Loftus et al., 2020; Uehara et al., 2023). Building on this work, we sought to determine whether osteoblast functional responses following tumor-derived EV enriched conditioned media exposure are conserved in the context of ER+ human breast cancer cells. To identify soluble mediators associated with tumor-bone interactions, we next profiled cytokines secreted in media collected from OBs treated with T47D EV-enriched conditioned media for 24 h in comparison to TBBM as a control (Supplementary Table 1). Out of the fourteen analytes, expression levels of SDF-1/CXCL12, VEGF-A, VEGF-C, and VEGF-D were significantly different in response to T47D-derived EV-enriched conditions compared to control conditions (Figure 4A). Stromal cell-derived factor-1 (SDF-1/CXCL12) is an osteoblast-derived chemokine that plays a central role in CXCR4-mediated signaling during bone development and cancer progression (Jung et al., 2006; Shahnazari et al., 2013; Sun et al., 2014). Following treatment with T47D EV-enriched media, osteoblasts exhibited a significant increase in SDF-1/CXCL12 expression. In addition, the angiogenic growth factor VEGF-A and the lymphangiogenic growth factor VEGF-C were both upregulated in response to T47D EV-enriched conditions. In contrast, expression of the lymphangiogenic growth factor VEGF-D was reduced following co-culture under T47D EV-enriched conditions (Figure 4A). To examine whether T47D EV treatment impacted osteoblast proliferation, we performed a proliferation assay on OBs cultured with T47D-derived EV-enriched conditioned media compared to TBBM control media. In response to T47D EV-enriched conditions, OBs were significantly more proliferative than OBs in TBBM (Figures 4B,C). As an indirect measure of proliferation, a wound healing assay was conducted to visualize whether EV-enriched conditions affected motility or rapid cell growth. Consistent with the proliferation data, we observed complete scratch closure of OBs following 24 h of T47D EV culture compared to TBBM control conditions (Supplementary Table 2; Supplementary Figure 2). To further evaluate that the OB proliferative response was specific to tumor-derived signaling, OBs were also treated with EV-enriched conditioned and naïve media from non-tumorigenic MCF10A cells. Compared with T47D EV-enriched conditioned media, OBs exposed to MCF10A EV-enriched conditioned media exhibited lower proliferation (Figure 4C). Moreover, OB proliferation was significantly reduced in MCF10A EV-enriched conditioned media relative to the corresponding control condition (Figure 4C).

**FIGURE 4:**
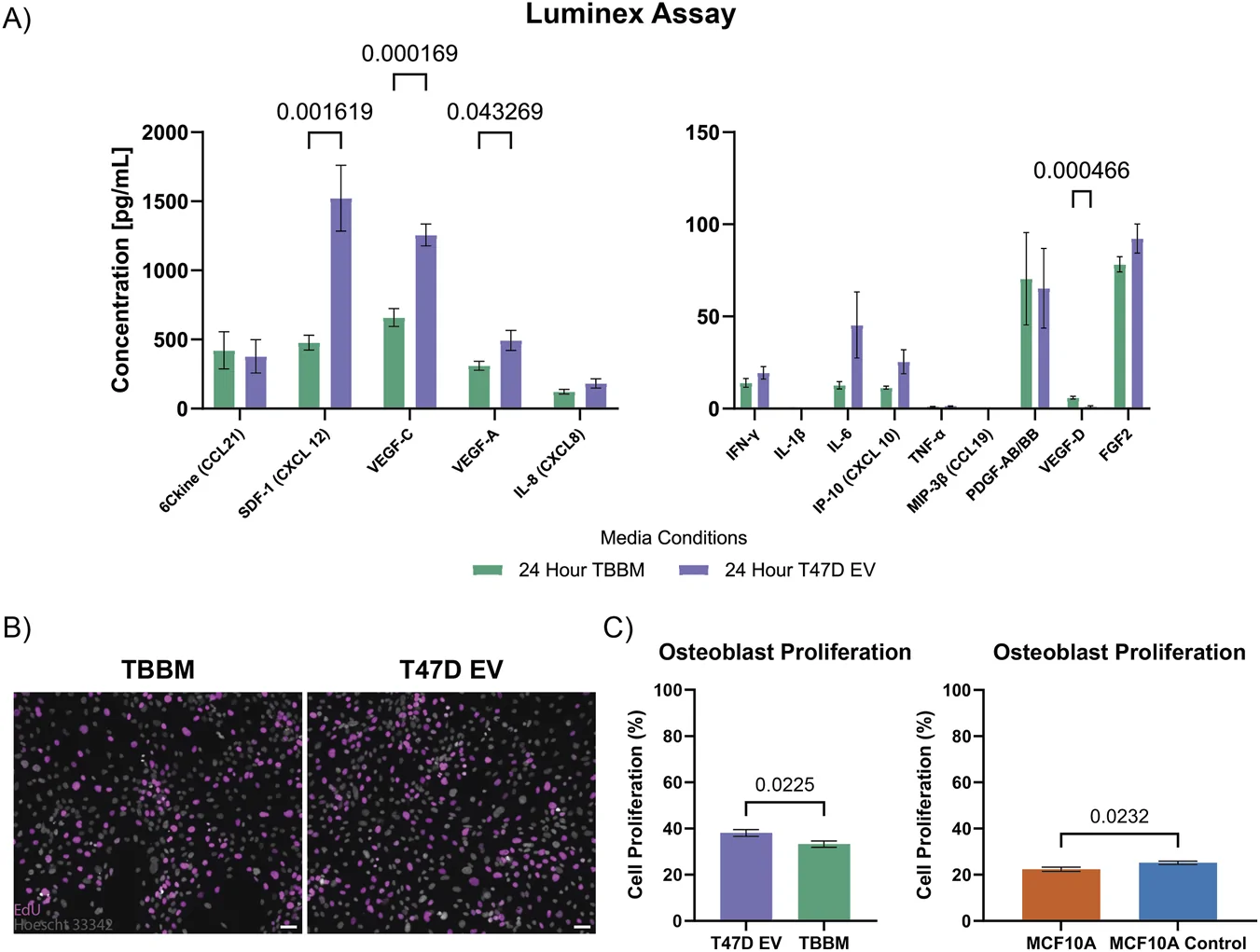
T47D EVs promote soluble factor secretion and osteoblast proliferation. **(A)** Quantification of soluble factor secretion from preconditioned OBs following 24 h exposure to T47D EV-enriched conditioned media. Analytes were run concurrently, and representative histograms are separated for appropriate data visualization via y-axis scaling. Statistical significance was determined via unpaired t-tests and with reported raw p-values shown that were considered significant following Benjamini, Krieger, and Yekutieli FDR correction (Q = 5%). **(B)** Representative images of proliferation of OBs cultured in media conditions TBBM (left) and T47D EV- enriched conditioned media (right). EdU-positive staining represents proliferative cells (magenta) and Hoescht 33342 represents total cells present (gray). Scale bar represents 50 µm. **(C)** Quantification of OB proliferation in response to TBBM and T47D EV-enriched conditioned media. Statistical significance was determined via unpaired Student’s t-test. Bar graph represents Mean ± SEM.

### 3.5 Preconditioning of osteoblasts with T47D-EV-enriched conditioned media produced a modest impact on T47D motility with limited effects on proliferation

T47D cell proliferation was not significantly affected by conditioned media from T47D-derived EV-preconditioned OBs (Supplementary Figure 3). Similarly, conventional mean-based migration metrics did not reveal any uniform changes in T47D cell motility. Visualization of single-cell trajectories using rose plots showed no overt directional bias across conditions, indicating that OB preconditioning did not induce directional migration of T47D cells (Figure 5A). Consistently, quantification of normalized average speed, total displacement, and net displacement revealed no statistically significant differences in mean values between groups (Figures 5B–D), suggesting that bulk motility metrics were not substantially altered.

**FIGURE 5:**
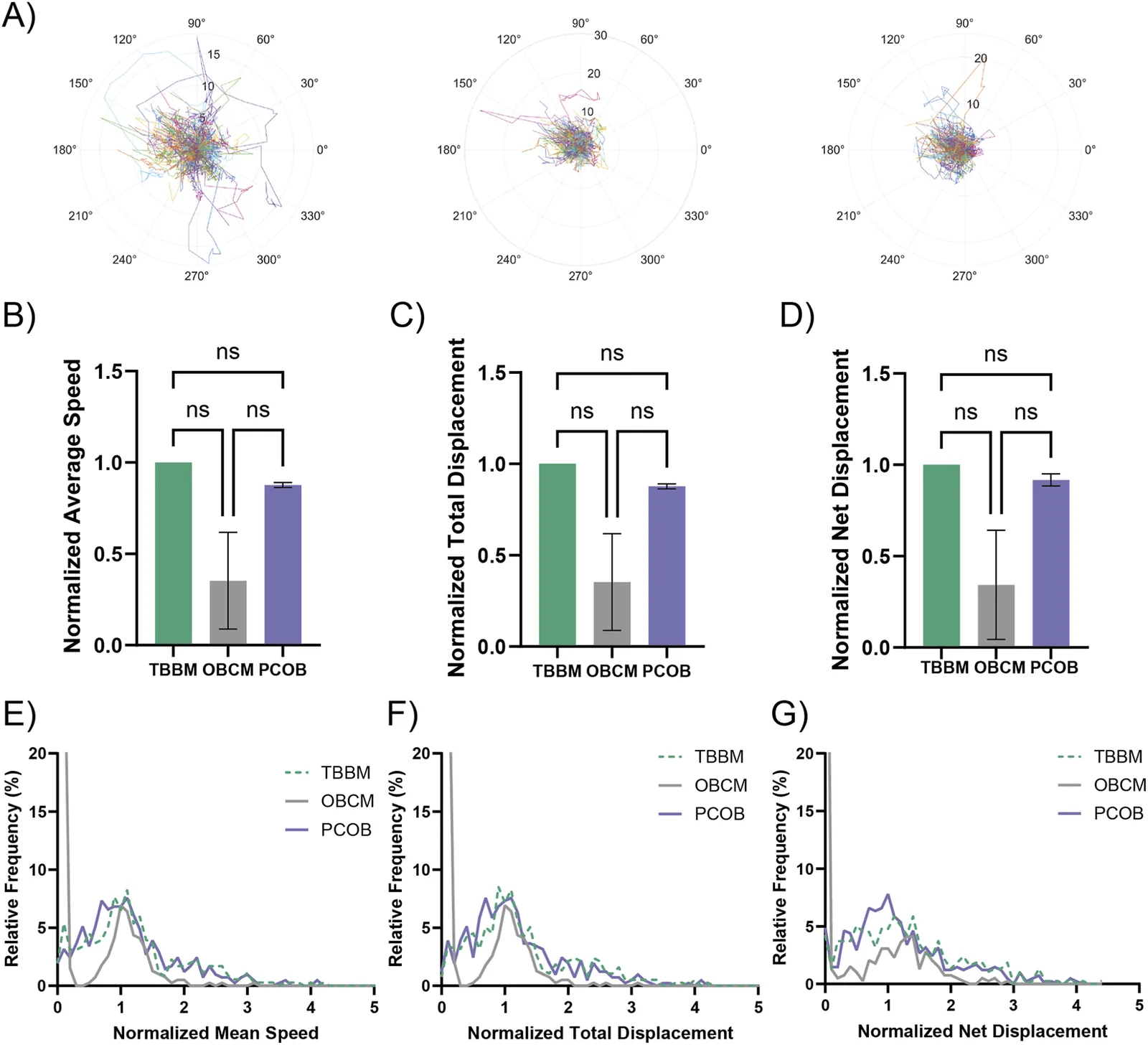
Preconditioning of osteoblasts with T47D-Derived EVs Shifts Breast Cancer Cell Motility Distributions. **(A)** Representative rose plots of single-cell trajectories under TBBM (left), OBCM (middle), and PCOB (right) conditions. **(B–D)** Quantification of normalized average speed **(B)**, total displacement **(C)**, and net displacement **(D)** (n = 3). Bar graph represents Mean ± SEM. Statistical analysis was performed via one-way ANOVA. n.s. indicates not significant. **(E–G)** Distribution plots of normalized mean speed **(E)**, total displacement **(F)**, and net displacement **(G)**, revealing population-level shifts in cell motility.

In contrast, distribution-based analyses revealed significant shifts in T47D cell motility profiles. For normalized speed, a significant overall effect was observed (Kruskal–Wallis test, p = 0.027), with Dunn’s multiple comparisons identifying differences between TBBM and OBCM-treated cells. These changes were supported by both the Kolmogorov–Smirnov test (p = 0.033) and the Mann–Whitney U test (p = 0.0069), indicating a redistribution towards lower motility within the OBCM-treated population (Figure 5E). Similarly, distribution-level effects were observed for total displacement, with significant differences (Kruskal–Wallis, p = 0.026). Post hoc testing revealed differences between TBBM and OBCM (Dunn’s multiple comparisons, p = 0.035). Distribution-level differences were further supported by the Kolmogorov–Smirnov test (p = 0.045) and Mann–Whitney U tests (PCOB vs. OBCM, p = 0.032; OBCM vs. TBBM, p = 0.0090) (Figure 5F). The strongest effects were observed for net displacement (Kruskal–Wallis, p = 0.013) (Figure 5G), where multiple statistical tests consistently indicated that OBCM treatment shifted the population toward reduced displacement with Dunn’s multiple comparisons identifying differences between TBBM and OBCM (p = 0.0019) and between OBCM and PCOB (p = 0.015). These findings were supported by Kolmogorov–Smirnov tests (TBBM vs. OBCM, p = 0.0033; OBCM vs. PCOB, p = 0.026) and Mann–Whitney U tests (PCOB vs. OBCM, p = 0.0066; OBCM vs. TBBM, p = 0.0030). Collectively, these findings indicate that OB preconditioning with T47D-derived EV-enriched conditioned media does not uniformly suppress T47D cell migration but instead alters the distribution of migratory behaviors across the cell population. This suggests that EV-mediated OB reprogramming influences T47D cell behavior in a heterogeneous manner, affecting specific subpopulations rather than producing uniform changes across the entire cell population.

## 4 Discussion

In this study, we investigated how ER+ breast cancer-derived EVs influence osteoblast behavior and signaling as a model of tumor-bone intercellular communication during breast-to-bone metastasis. Intercellular communication between breast tumor cells and bone-resident stromal cells is a key driver of premetastatic progression (Müller et al., 2001; Allocca et al., 2019; Zarrer et al., 2020; Song et al., 2022; Xu et al., 2024; Ma et al., 2025). Upon dissemination, tumor cells localize to the bone marrow niche where they encounter osteoblasts within the endosteal niche as some of the earliest stromal cell interactors (Haider et al., 2020). These initial tumor-osteoblast interactions occur prior to overt metastatic lesion formation and are increasingly recognized as essential for the establishment of a supportive microenvironment (Reagan and Rosen, 2015) that facilitates subsequent colonization and disease progression by influencing osteoclast activation (Ottewell, 2016; Wang et al., 2020) While soluble breast cancer-derived factors have been widely studied as mediators of tumor-bone signaling (Evans et al., 1991; Lee et al., 2017), EV-mediated communication represents an additional and comparatively underexplored mechanism through which tumor cells may reprogram surrounding stromal populations in bone. Notably, EVs are known mediators of premetastatic niche formation, highlighting their potential importance in shaping early stages of bone metastasis.

Previous studies investigating ER+ breast cancer-bone interactions have frequently employed MCF7 cells, demonstrating ER+ breast cancer cells engage in reciprocal signaling with osteoblasts and other associated cell populations to promote tumor adaptation within the bone niche (Nicolin et al., 2008; Arrigoni et al., 2016). However, whether these mechanisms extend across additional ER+ breast cancer models remains incompletely understood. Despite the clinical relevance of T47D as a well-established luminal A molecular subtype breast cancer model (Pink et al., 1996; Yu et al., 2017), mechanistic studies examining T47D-osteoblast interactions remain limited. Characterizing communication between T47D cells and osteoblasts therefore expands our understanding of tumor-bone crosstalk in ER+ breast cancer and broadens the range of experimental models used to study breast to bone metastasis.

Further, emerging evidence in breast-to-bone metastasis demonstrates that breast tumor-derived EVs regulate osteoblast function and indirectly influence osteoclast activity, thereby shaping bone remodeling dynamics. In combination with soluble factor communication, comprehensive understanding of how EVs contribute to bone premetastatic niche formation will provide insights on new opportunities for therapeutic intervention. As early stromal interfaces for disseminated breast tumor cells, osteoblasts represent a critical cell population through which tumor-derived EVs may shape early metastatic programming. In this work, we demonstrate that osteoblasts respond to ER+ tumor-derived EV-enriched conditions. We first characterized EVs released from ER+ T47Ds, where we found that they have increased EV production rate compared to non-tumorigenic MCF10A breast epithelial cells, consistent with prior findings, including our own, that non-transformed epithelial cells display lower EV output (Antonyak et al., 2011; Shurer et al., 2019). This increase was most pronounced within the MV size range (specifically 150–500 nm), where the maximum production rate was significantly elevated. Notably, we have previously demonstrated that MV biogenesis within this size regime is associated with enhanced plasma membrane bending, a process linked to increase in glycocalyx biopolymer expression in cancer cells (Shurer et al., 2019; Noble et al., 2020).

We next evaluated osteoblast uptake dynamics and downstream signaling responses following exposure to EV-enriched conditioned media from human T47D breast tumor cells. Consistent with our previously validated protocol, EVs were robustly enriched within conditioned media preparations (Shurer et al., 2019; Noble et al., 2020). While these EV-enriched conditions may contain co-existing soluble factors, the observed response patterns are consistent with EV uptake by osteoblasts. Moreover, the relatively short half-life of soluble VEGF family members, particularly VEGF-A (Beheshtizadeh et al., 2023), may suggest that the observed increases reflect osteoblast-mediated signaling responses within EV-enriched conditions rather than persistent accumulation of tumor-derived soluble factors. Nanoparticle tracking analysis revealed a progressive decrease in EV particle concentration at 6, 12, and 24 h, demonstrating active uptake of T47D-derived EVs by OBs over time. In contrast, OBs cultured in TBBM control conditions exhibited lower EV baseline levels and did not display significant changes in particle concentration at time points. EV uptake was further confirmed by fluorescence microscopy using MemGlow-labeled EVs, supporting a direct role for EV-mediated modulation of OB behavior.

Profiling of cytokines revealed that EV-enriched conditions increased expression of SDF-1/CXCL12, VEGF-A, and VEGF-C, alongside a decreased expression of VEGF-D. Osteoblasts are key regulators of the bone marrow niche and contribute to tumor cell homing through the secretion of SDF-1/CXCL12 (Huo et al., 2025). This signaling axis promotes chemotactic cues within the bone microenvironment and facilitates tumor cell recruitment via the well-established SDF-1/CXCR4 axis (Haider et al., 2020; Dai et al., 2022; Liu et al., 2022; Huo et al., 2025), reinforcing the significance in our findings. However, through our motility studies, we did not observe significantly enhanced motility as expected. In cancer, tumor cells are in close proximity to osteoblasts and highly implicated in directing tumor cells into a dormant state (Zarrer and Taipaleenmäki, 2024), which could explain why we did not see any changes in tumor cell motility or it could be context dependent.

In parallel, VEGF-A expression was increased, consistent with its well-established secretion by osteoblasts and its known role in tumor-associated angiogenic remodeling (Ferrara, 2004; Zarrer et al., 2020; McKenzie et al., 2022). Similarly, we also observed upregulation of VEGF-C, which is associated as a lymphangiogenic growth factor that is more commonly attributed to tumor-derived signaling and metastatic dissemination (Su et al., 2006; Timoshenko et al., 2007; Chen et al., 2012; Chen et al., 2019; Varney and Singh, 2015; Tzeng et al., 2016) rather than baseline osteoblast secretion. While VEGF-A has been extensively studied in the context of osteoblast biology, comparatively few studies have examined the contribution of additional VEGF family members (Deckers et al., 2000). Prior work suggests that osteoblasts do not express VEGF-C under basal conditions; however, VEGF-C regulation in osteoblasts under tumor-associated or pathological conditions remains largely unexplored. Given emerging evidence of lymphangiogenesis in bone remodeling and repair (Hominick et al., 2018), future studies examining VEGF-C regulation in osteoblasts under tumor-associated conditions will provide insight into mechanisms linking lymphatic remodeling with metastatic progression and vascular adaptation within the tumor-supportive bone niche. Importantly, VEGF signaling exhibits pleiotropic effects with prior studies demonstrating that VEGF-A can promote breast tumor cell proliferation, survival, adhesion, migration, and chemotaxis through angiogenesis-independent autocrine signaling mechanisms (Perrot-Applanat and Di Benedetto, 2012). These functions are particularly relevant in the context of metastatic dormancy (Zarrer and Taipaleenmäki, 2024), as bone-disseminated tumor cells may persist in a non-detectable state before transitioning toward proliferative outgrowth with angiogenic remodeling supporting dormancy-mediated treatment resistance (Mayhew et al., 2019). Thus, EV-induced regulation of VEGF-A and VEGF-C by osteoblasts may contribute not only to remodeling of the vascular and lymphatic niche, but also to survival- and growth-associated signaling programs that support dormant or reactivating tumor cells. Collectively, our findings indicate that osteoblasts exposed to EV-enriched conditioned media adopt a tumor-educated phenotype, characterized by functional reprogramming that would disrupt bone homeostasis to promote tumor-supportive behavior (Kolb et al., 2019; Huang et al., 2022; Coronado-Alvarado and Astiazaran-Garcia, 2025).

Interestingly, VEGF-D expression was reduced following exposure to T47D EV-enriched conditions. VEGF-D has been reported to be expressed in osteoblasts within the long bone and, via signaling through VEGFR3, is a key lymphangiogenic receptor with established roles in osteoblast differentiation and skeletal development (Orlandini et al., 2006). The observed reduction suggests that EV-mediated signaling does not induce a global increase in pro-vascular factors but instead drives selective pathway regulation. While VEGF-D has also been implicated in lymphatic remodeling and metastasis (Karnezis et al., 2012), its reduction suggests differential control within the VEGF signaling network. This finding points toward a more refined mechanism of osteoblast reprogramming, in which tumor-derived EVs selectively activate signaling pathways that favor tumor progression rather than broadly amplifying all pro-metastatic signals. Although not significant, IL-6 expression was higher with EV treatment, which is important as it is associated with osteoclastogenesis and tumor growth (Sethi et al., 2011). These signaling changes were accompanied by increased osteoblast proliferation following EV exposure, supporting the concept of an activated osteoblast phenotype. Interestingly, we found that osteoblast proliferation was reduced in response to MCF10A EV-enriched conditioned media relative to the control condition, whereas T47D EV-enriched conditioned media increased proliferation. These findings support that osteoblast proliferative responses differ between non-tumorigenic and tumor-derived EV-enriched conditions. Increased osteoblast proliferation may contribute to disruption of normal bone homeostasis and expansion of a tumor-supportive niche, consistent with prior findings demonstrating that breast tumor cells can reprogram osteoblasts to promote tumor growth and microenvironmental conditioning (Kolb et al., 2019). In contrast to other studies reporting that reduced osteoblast activity and proliferation was reduced under tumor-associated conditions (Loftus et al., 2020; Dabaliz et al., 2025), the responses observed in our findings may be context-dependent and influenced by cancer cell type or molecular subtype. Systematic comparisons across multiple breast tumor cell lines representing distinct molecular subtypes will be necessary to determine the degree to which osteoblast responses vary with tumor identity. In addition, future studies profiling the molecular cargo of T47D-derived EVs, as well as EVs released from tumor preconditioned osteoblasts, may provide further insight into EV-associated signaling pathways that contribute to osteoblast modulation and bone metastatic niche formation.

Finally, we examined whether osteoblasts preconditioned by tumor-derived EV-enriched conditioned media communicate back to T47D cells. Osteoblast-derived EVs have been shown to deliver bioactive molecules capable of influencing bone remodeling and cell behavior (Cappariello et al., 2018; Niedermair et al., 2020; Uenaka et al., 2022; Davies, 2023), with prior studies suggesting osteoblast-conditioned media can enhance tumor cell motility (Chen et al., 2014; Shi et al., 2021). In contrast, we observed no significant differences between mean migration speed, chemotactic index, nor total displacement across conditions. However, analysis of relative cell behavior distributions revealed differences in the frequency of specific migratory patterns, suggesting that preconditioned osteoblast-derived EV-enriched conditioned media may subtly influence migration behavior without producing uniform changes in global motility outcomes. Future studies examining phenotypic heterogeneity of responsive tumor cell subpopulations and molecular cargo of osteoblast-derived EVs may help clarify these distribution-level effects. Overall, our findings demonstrate that EV-enriched conditions influence osteoblast behavior and signaling, providing insight into how preconditioning of bone stromal cells may influence early mechanisms underlying lesion formation in breast-to-bone metastasis.

Despite the strengths of this study, several limitations should be acknowledged. First, this work was designed to isolate osteoblast responses to tumor-derived EVs; however, the endosteal niche comprises of a heterogeneous bone-cell population, including osteoclasts and osteoprogenitor cells. Future studies should incorporate these cell types, either individually or in combination, to better characterize cell-cell interactions and signaling pathways associated with metastatic lesion development. Second, experiments were conducted under static conditions to isolate the effects of tumor-derived EVs on osteoblasts. However, the *in vivo* metastatic niche is inherently dynamic, with signaling regulated by the transport of cells, extracellular vesicles, and soluble factors that cannot be fully captured under static culture conditions. Accordingly, future studies should utilize *in vitro* models that incorporate physiologically relevant transport conditions to evaluate their effects on tumor-stromal communication and behavior. Given the observed modulation of lymphangiogenic factors under EV-enriched conditions, inclusion of lymphatic endothelial cells is also warranted, as their role in metastatic progression remains underexplored yet likely significant. Third, this study characterized preconditioned osteoblasts through EV uptake and cytokine profiling. Future work should extend culture durations to determine whether prolonged exposure leads to further alterations in secretory profiles (e.g., particle size distribution, concentration, or downstream growth factor expression), which may better reflect long-term microenvironmental remodeling and late-stage metastatic progression. Lastly, the concentration of growth factors and cytokines within T47D-derived EVs was not quantified in the present study. Rather, our analyses focused on the downstream effects of EV exposure on osteoblast secretory signaling. Recent advances have demonstrated the feasibility of characterizing the protein and cytokine cargo contained within EVs (Feng et al., 2017; Treps et al., 2017; Dalla et al., 2020), providing valuable insight into the molecular mediators responsible for EV-induced cellular responses. Therefore, future studies should incorporate EV lysis and cargo characterization to identify and quantify growth factors, cytokines, and other bioactive molecules carried by T47D-derived EVs and to better define the mechanisms underlying osteoblast reprogramming.

Overall, we demonstrate that EVs derived from the ER+ breast cancer cell line promote osteoblast reprogramming characterized by altered proliferation and signaling, supporting EV-mediated tumor-osteoblast communication as an important contributor to early metastatic niche conditioning. Despite the complexity of the bone microenvironment, our findings highlight osteoblasts as important intermediaries in EV-mediated preconditioning of the metastatic niche. Future studies defining EV-associated cargos, stromal signaling pathways, and multicellular interactions will be critical for advancing predictive biomarkers and therapeutic strategies for breast-to-bone metastasis.

## Data Availability

The original contributions presented in the study are included in the article/Supplementary Material, further inquiries can be directed to the corresponding authors.
