# Supplementary material for: Extracellular vesicle-mediated preconditioning of osteoblasts by ER+ breast cancer cells: implications for the bone metastatic niche
Source: Front Pharmacol. 2026 Sep 28;17:1844422. doi: 10.3389/fphar.2026.1844422 (PMC13656745; doi:10.3389/fphar.2026.1844422)
Supplement: Supplementary file 1 [file DataSheet1.pdf]

Supplementary Material

1 Supplementary Methods

1.1 T47D Proliferation Assay

Proliferation was quantified using the Click-iT™ EdU cell proliferation assay (Invitrogen, C10340) as previously described. For T47D proliferation studies following culture with conditioned media from OBs treated with EVs, 1.9 x 10^4 T47Ds were seeded in 24-well dishes and treated with either TBBM, OB conditioned media (OBCM), or EV pre-conditioned OBCM (PCOB). After 18 h of culture, 10 µM EdU was added to media and incubated for 6 h. Subsequently, cells were washed with PBS and fixed with 4% paraformaldehyde. Samples were then permeabilized with 0.5% Triton X-100 and stained with the Click-iT™ reaction cocktail per the manufacturer's instructions. The cells were then counterstained with Hoescht 33342 or DAPI, targeting the nucleus, at a concentration of 5 µg/mL. Once stained, five regions of interest (ROI) were imaged across each well under 10X magnification using the Discover ECHO Revolution microscope. A custom CellProfiler pipeline was used to identify and quantify DAPI-positive nuclei. EdU-positive nuclei were then detected using the same segmentation framework to evaluate the labeled cell population.

1.2 Scratch/Wound Healing Assay

1.5 x 10^5 OBs were seeded in 24-well dishes and cultured until a confluent monolayer was established as described above. Once the monolayer formed, a vertical scratch was made by a P200 pipette tip. Released cells were aspirated and washed with PBS. After the scratch was washed, OBs were treated with either T47D EV-enriched conditioned media or TBBM media as a control. Based on preliminary data, wound healing was not observed within the first 6 h; therefore, to optimize equipment utilization while maintaining data integrity, time-lapse live imaging was performed with images acquired every hour for the last 18 h using the EVOS M7000 onstage incubator imaging system at 37°C and 5% CO2 for a total of 24 h.

1.3 Image Quantification

For wound healing, closure was quantified using a line measuring macro plugin in Fiji (ImageJ). Closure was determined by measuring the distance between the two wound edges across time points (0, 6, 12, and 24 hours), with multiple fields of view analyzed per well. Scratch closure is represented by percentage closure, which is calculated by averages and standard deviation. Publicly available ImageJ macro code by Santosh Patnaik, MD, PhD was used to measure scratch width.

2 Supplementary Figures and Tables

2.1 Supplementary Tables

Supplementary Table 1. Concentrations of OB secreted growth factors and cytokines following 24 h co-culture of TBBM and EV-conditioned media. Quantification is represented by average concentrations and standard deviation (n = 3).

Makers	TBBM (pg/ mL)	T47D EVs (pg/ mL)
IFN-γ	17.77 ± 8.10	13.91 ± 5.80
IL-1β	n.d.	n.d.
IL-6	45.37 ± 43.91	12.68 ± 4.88
IP-10 (CXCL 10)	25.40 ± 15.93	11.37 ± 1.88
PDGF-AB/BB	65.31 ± 52.97	70.47 ± 61.37
TNF-α	1.27 ± 0.37	1.07 ± .028
6Ckine (CCL21)	378.24 ± 294.19	421.40 ± 329.10
MIP-3β (CCL19)	n.d.	n.d.
**SDF-1 (CXCL 12)**	**1523.02 ± 584.04**	**477.89 ± 131.02**
IL-8 (CXCL8)	182.66 ± 82.55	122.35 ± 40.51
**VEGF-C**	**1256.04 ± 194.42**	**659.27 ± 159.08**
**VEGF-D**	**0.96 ± 1.37**	**5.93 ±1.95**
FGF2	92.32 ± 19.38	78.33 ± 10.13
**VEGF-A**	**439.79 ± 493.73**	**310.17 ± 81.21**

n.d. is not detected
Bolded values indicate significance ($p < 0.05$)

Supplementary Table 2. OB scratch closure from 24 h co-culture of TBBM and T47D EV-conditioned media. Quantification represents average percentage closure and standard error (n=2).

Conditions	Percent Closure (%)
TBBM (0h)	0
TBBM (6h)	34.83 ± 1.53
TBBM (12h)	54.97 ± 1.70
TBBM (24h)	81.33 ± 2.77
EV-conditioned (0 h)	0
EV-conditioned (6h)	31.12 ± 1.36
EV-conditioned (12h)	66.16 ± 2.46
EV-conditioned (24h)	100

2.2 Supplementary Figures

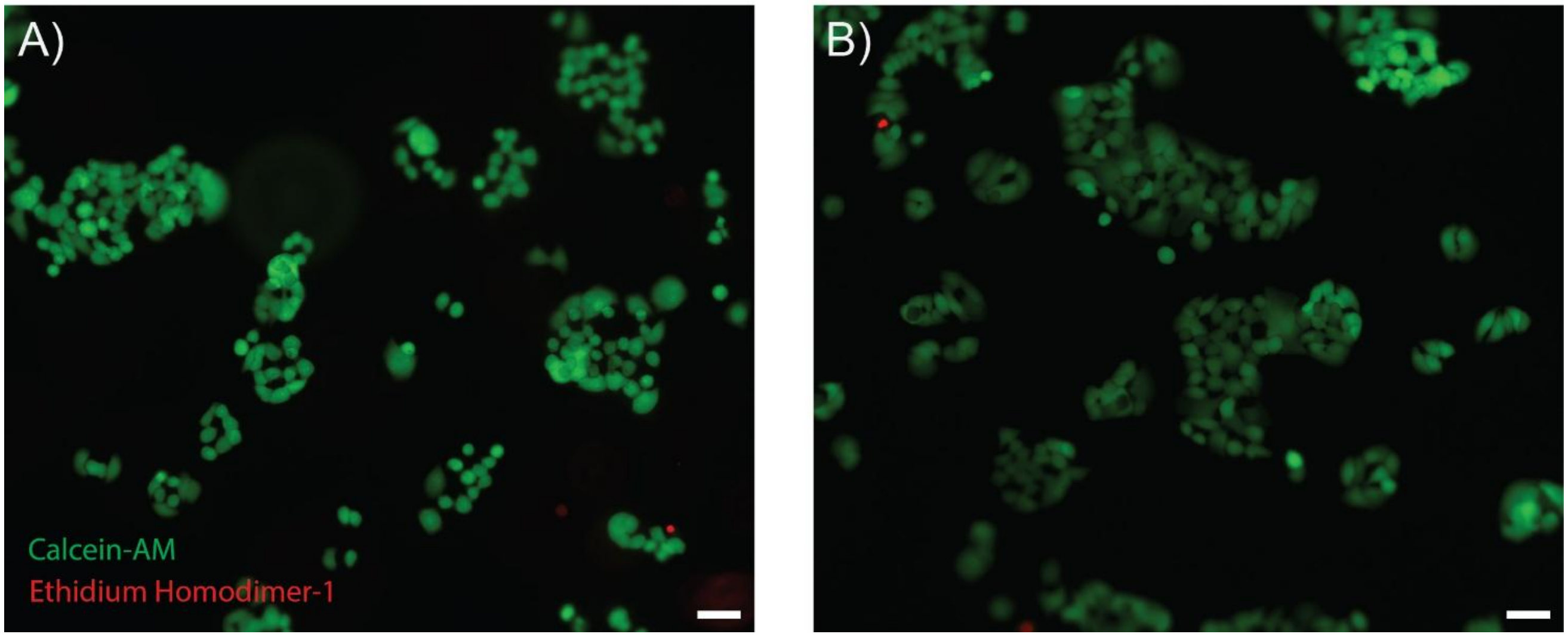

Supplementary Figure 1. T47D viability is not affected in osteogenic media conditions. Representative images of T47D viability following culture in **(A)** T47D media and **(B)** osteogenic media after 24 h. Live cells are denoted in green and dead cells are denoted in red. Scale bar represents 50 µm.

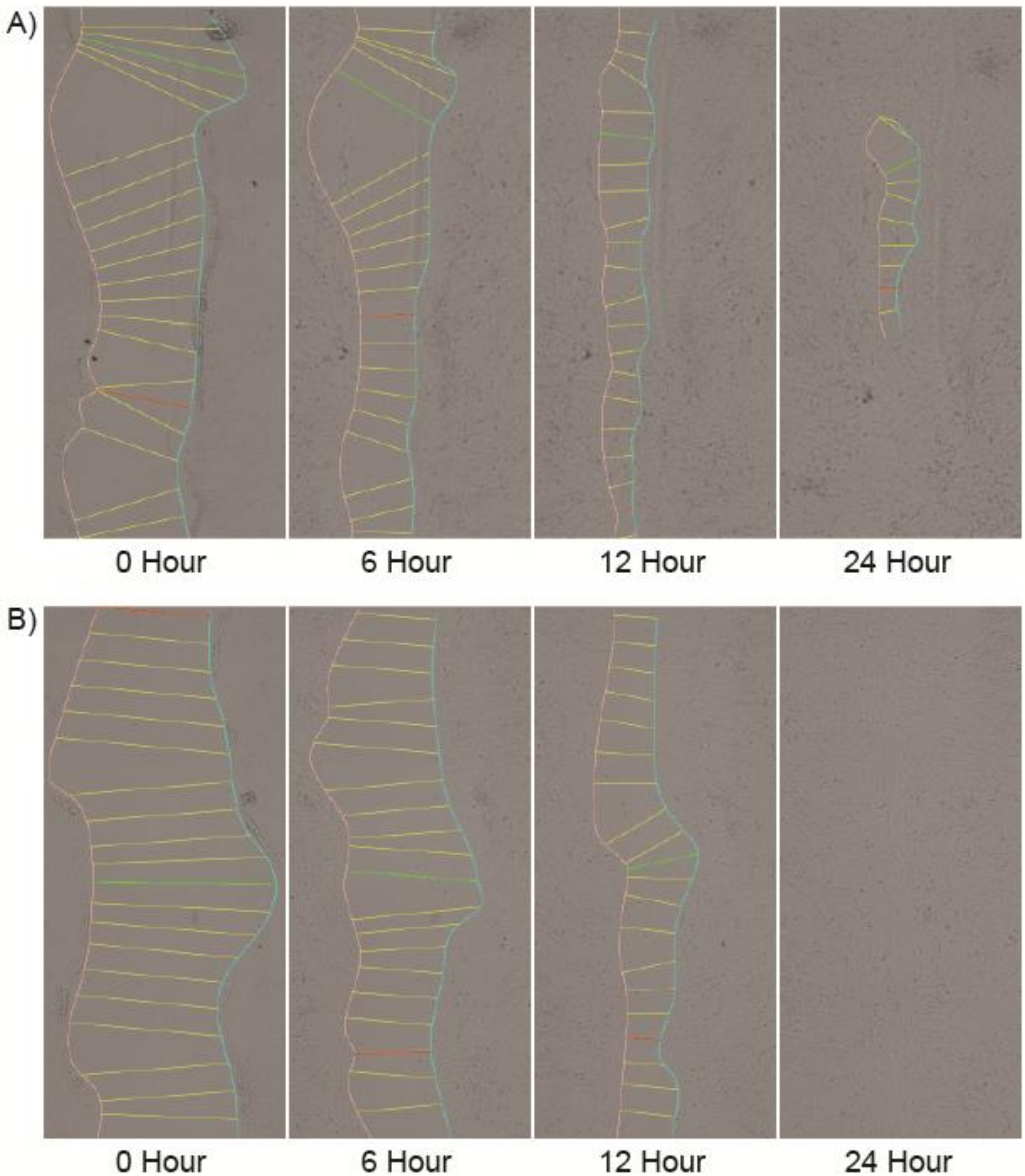

Supplementary Figure 2. T47D-enriched conditions result in complete osteoblast scratch closure compared to controls after 24 h. Representative images of scratch closure of **(A)** OBs supplemented with TBBM and **(B)** T47D EV-conditioned media at 0, 6, 12, and 24 h (n = 2).

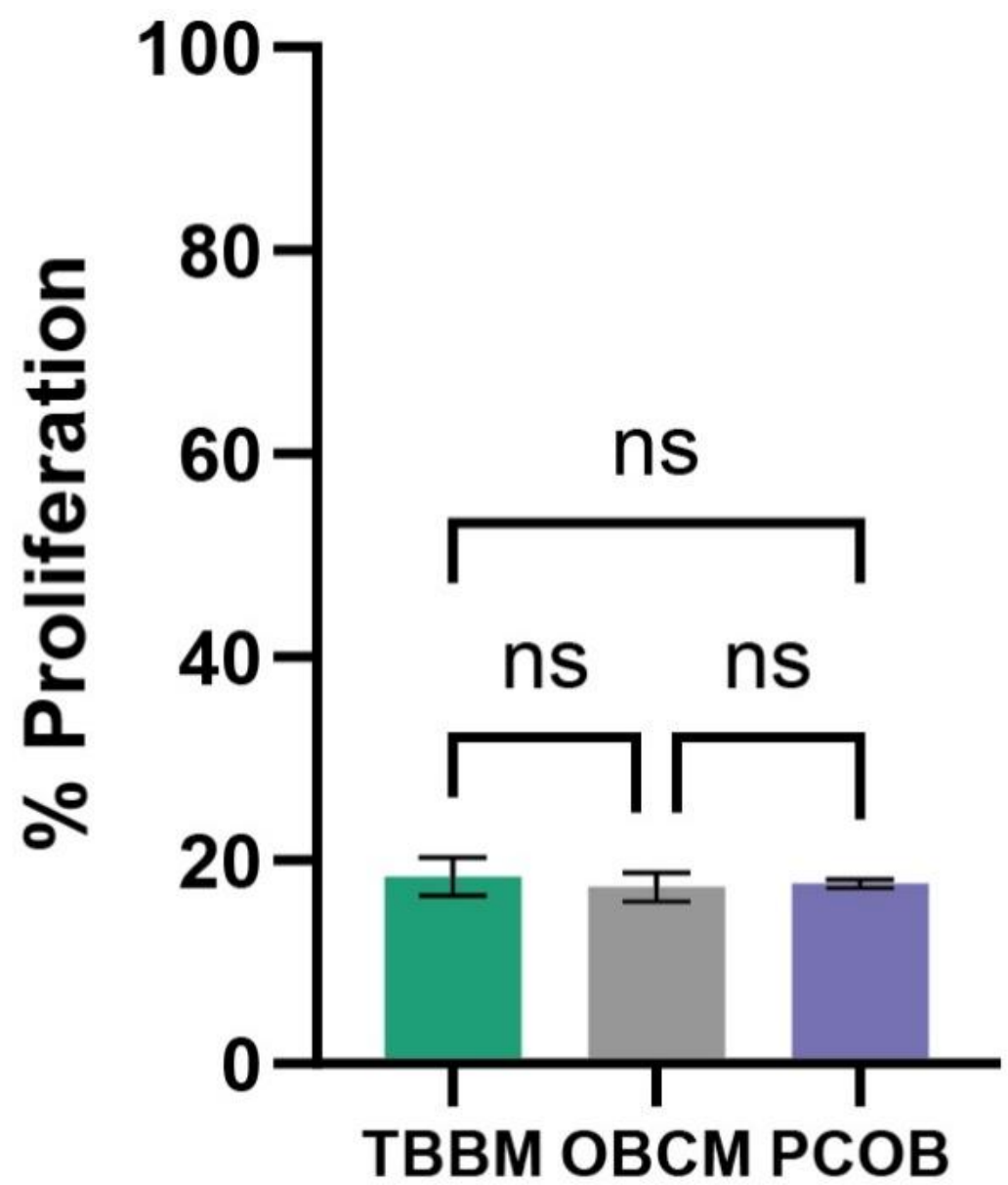

Supplementary Figure 3. T47D proliferation was not altered when co-cultured with osteoblast conditioned media (OBCM) nor pre-conditioned osteoblast media (PCOB) compared to control. Quantification of T47D proliferation in response to TBBM, OBCM and PCOB. Statistical significance was determined via unpaired Student's *t*-test. Bar graph represents Mean ± SEM (n = 3).
